# An Approach to Assess Generalizability in Comparative Effectiveness Research: A Case Study of the Whole Systems Demonstrator Cluster Randomized Trial Comparing Telehealth with Usual Care for Patients with Chronic Health Conditions

**DOI:** 10.1177/0272989X15585131

**Published:** 2015-11

**Authors:** Adam Steventon, Richard Grieve, Martin Bardsley

**Affiliations:** Health Foundation, London, UK (AS); London School of Hygiene and Tropical Medicine, Keppel Street, London (AS, RG); Nuffield Trust, London (MB)

**Keywords:** causal inference, external validity, generalizability, randomized trials, telehealth, chronic health conditions

## Abstract

**Background.** Policy makers require estimates of comparative effectiveness that apply to the population of interest, but there has been little research on quantitative approaches to assess and extend the generalizability of randomized controlled trial (RCT)–based evaluations. We illustrate an approach using observational data. **Methods.** Our example is the Whole Systems Demonstrator (WSD) trial, in which 3230 adults with chronic conditions were assigned to receive telehealth or usual care. First, we used novel placebo tests to assess whether outcomes were similar between the RCT control group and a matched subset of nonparticipants who received usual care. We matched on 65 baseline variables obtained from the electronic medical record. Second, we conducted sensitivity analysis to consider whether the estimates of treatment effectiveness were robust to alternative assumptions about whether “usual care” is defined by the RCT control group or nonparticipants. Thus, we provided alternative estimates of comparative effectiveness by contrasting the outcomes of the RCT telehealth group and matched nonparticipants. **Results.** For some endpoints, such as the number of outpatient attendances, the placebo tests passed, and the effectiveness estimates were robust to the choice of comparison group. However, for other endpoints, such as emergency admissions, the placebo tests failed and the estimates of treatment effect differed markedly according to whether telehealth patients were compared with RCT controls or matched nonparticipants. **Conclusions.** The proposed placebo tests indicate those cases when estimates from RCTs do not generalize to routine clinical practice and motivate complementary estimates of comparative effectiveness that use observational data. Future RCTs are recommended to incorporate these placebo tests and the accompanying sensitivity analyses to enhance their relevance to policy making.

Well-conducted randomized controlled trials (RCTs) can ensure high levels of internal validity because the treatment groups are balanced. However, a major concern with RCT evidence is that the resultant estimates may not generalize directly to the target population of interest.^1^ Even if an RCT has a pragmatic design without restrictive inclusion criteria and compares the intervention with usual care,^2^ the trial may exclude important subgroups of patients and centers.^3^ Thus, both observed and unobserved characteristics that modify treatment effects may differ between the RCT participants and the target population. Another threat to generalizability is that the care provided in the RCT may differ from what would be delivered in routine clinical practice. These concerns about generalizability explain why technologies shown to be beneficial in RCTs may not be diffused into routine practice.^4^

Observational studies, such as prospective cohort studies, have the potential to include a broad range of patients, settings, and treatment options, representative of those in routine practice. However, in attempting to estimate comparative effectiveness from observational studies, the major methodological challenge is confounding due to treatment selection.^5,6^ While there have been recent improvements in methods to deal with confounding,^7,8^ these tend to assume that there is no unobserved confounding, which may be implausible.^9^ Rather than regarding RCTs and observational studies as mutually exclusive alternatives, a promising research agenda has emerged that uses observational data to assess the generalizability of RCT evidence.^6,10–15^

Cole and Stuart^6^ described how treatment effects from an RCT could be generalized by reweighting them to reflect the characteristics of patients in the target population. Stuart and colleagues^12^ proposed a diagnostic test that assesses whether patients receiving a given treatment in an RCT reported similar outcomes to those receiving the same treatment in the target population, after reweighting for the characteristics of the 2 groups. Hartman and colleagues^14^ formally defined the assumptions required for estimating population treatment effects from RCT data and proposed accompanying placebo tests. None of this previous work examined how the study should proceed when the placebo tests fail to confirm the generalizability of an RCT. This is an important topic for policy makers, because if a placebo test fails, then it may be unclear whether the intervention is effective in the target population. In this paper, we extend placebo tests by proposing a sensitivity analysis that addresses the potential implications of nongeneralizability. We also apply placebo tests to an RCT of a complex, out-of-hospital intervention.^16^

Complex interventions are of particular interest to policy makers and include patient-centered medical homes,^17^ telehealth,^18^ and health coaching.^19^ Placebo tests have not previously been applied to RCTs of these interventions, but they face particular threats to generalizability.^16^ For example, the effectiveness of complex interventions is thought to depend on how well teams of care professionals work together, but the presence of an RCT can significantly alter the context in which these teams work.^20^ Also, patients and professionals often cannot be blinded to treatment allocations in these trials, since the control treatments (e.g., usual care) are typically already known to the participants, and the new interventions require changes in their behavior.^2^ There are several potential problems with estimating treatment effects from these unblinded trials, including “resentful demoralization” if a strong preference for the new treatment leads to poor compliance among controls.^21,22^

A clear example of the additional generalizability concerns that are raised by evaluations of complex interventions is the recent Whole Systems Demonstrator (WSD) trial, which used cluster randomization to assign 3230 patients with chronic conditions to receive either telehealth or usual care.^23^ In this trial, telehealth was associated with around 20% fewer unplanned (emergency) hospital admissions than usual care,^24^ which led to a national initiative to roll out telehealth and similar approaches to 3 million people in England.^25^ However, the estimated improvements in outcomes following telehealth appeared to be driven by the relative increase in rates of emergency admission among the RCT control group shortly after their recruitment,^20,24^ thus raising concern that patients in the control arm reacted to their allocations or received different care than would be provided in routine clinical practice.

Next we describe the WSD trial and discuss the generalizability concerns it illustrates. Then we describe placebo tests for assessing whether outcomes differ between the RCT control group and a matched sample from the target population, and apply these tests to the WSD trial. We propose sensitivity analyses to provide complementary treatment effects to address generalizability concerns. Finally, we discuss the implications for comparative effectiveness research.

## Running Example, the WSD Trial

The WSD trial used a pragmatic design to assess the impact of telehealth in the context of the routine delivery of care.^26^ All primary care practices within 3 study sites in England (Cornwall, Kent, and Newham) were eligible to participate; practices that accepted the invitation (*n* = 369) were randomized according to a minimization algorithm to provide either telehealth or usual care patients.^23^ Patient inclusion criteria were deliberately broad and specified only age 18 or over plus a diagnosis of chronic obstructive pulmonary disease (COPD), diabetes, or heart failure.

The trial was designed to detect a 17.5% relative change in hospitalization from a baseline of 25%, at 80% power and a 2-sided value of *P* < 0.05.^23^ The targeted number of patients was 3000. Potentially eligible patients were identified from the lists of patients registered at the participating primary care practices; diagnoses were sourced from routine primary and secondary care data sets and from clinician reports. Identified patients were written to at home (*n* = 15,171). Those who responded affirmatively (*n* = 5279) were visited and provided with consent forms for participation. Ultimately, 3230 patients participated. The treatment allocations of patients followed those of the primary care practices at which they were already registered. While patients could not be blinded, they were only told of their treatment allocations after they had consented to participate. The long recruitment period (May 2008 to September 2009) meant that it was not always possible to blind those recruiting patients.

Telehealth patients received home-based technology to record medical information (e.g., blood oxygen) and to answer symptom questions. Information from patients was transmitted automatically to monitoring centers, which were staffed by employees from local healthcare organizations. Control patients had access to usual care for their area, which did not include telehealth. They were offered telehealth at the end of the 12-month trial period if they were still eligible at that point.

For the analysis of service utilization, primary care practices were asked to share pseudonymized data from the electronic medical record for all their registered patients, covering dates of registrations, encounters, diagnoses, test results, and prescriptions over at least a 4-year period.^27^ These data were linked to pseudonymized administrative hospital records.^28^

In prespecified analyses, telehealth patients experienced fewer emergency hospital admissions than controls over 12 months (incidence rate ratio 0.81, 95% confidence interval [CI] 0.65–1.00, *P* = 0.046). Differences in other categories of healthcare utilization were not statistically significant; this included rates of planned (elective) admissions, emergency room visits, outpatient attendances, and primary care contacts.^24,27^ However, intervention patients experienced lower mortality than controls over 12 months. The relative difference, as measured by the odds ratio, was 0.54 (95% CI 0.39–0.75, *P* < 0.001), although the absolute change was relatively small (4.6% mortality for telehealth v. 8.3% for usual care).

Although selection bias is often a concern in cluster-randomized trials,^29^ no differences were detected between the baseline characteristics of telehealth and control patients, and effect sizes remained similar after adjustment, suggesting that internal validity was not a major issue. The evaluation protocol prespecified comparisons between RCT participants and nonparticipants to consider the generalizability concerns that we now discuss.

## Concerns about the Generalizability of the WSD Trial

**Figure 1:**
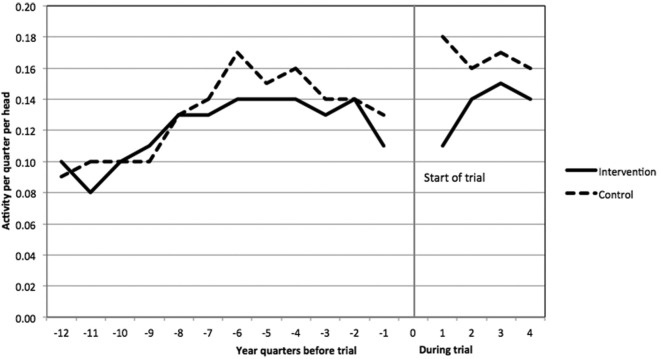
Patterns of emergency hospital admissions in the Whole Systems Demonstrator (WSD) telehealth trial (*n* = 3154). Figure reprinted with permission from Steventon A, Bardsley M, Billings J, et al. Effect of telehealth on use of secondary care and mortality: findings from the WSD cluster randomised trial. BMJ. 2012;344:e3874. http://www.bmj.com/content/344/bmj.e3874. Copyright © 2012, British Medical Journal Publishing Group.

Concerns about generalizability arose for several reasons. First, as is typical in telehealth trials,^30^ only a small proportion of the contacted patients agreed to participate in WSD (21.2%), suggesting that participants might be unrepresentative of the general population with chronic conditions.^31^ Second, emergency admission rates increased among control patients shortly after their recruitment (Figure 1),^24^ suggesting that these patients might not have received usual care. Finally, a qualitative study found that the trial protocol and recruitment processes hindered the participating sites’ attempts to develop integrated telehealth services,^20^ suggesting that telehealth might also differ between the RCT and routine practice.

Although the increase in emergency admissions could represent the normal evolution of need for healthcare among patients with chronic conditions, this seemed unlikely.^32^ Instead, healthcare professionals might have identified unmet need while recruiting patients and changed the management of those patients allocated to the control group, compared with usual care.^24^ Alternatively, the trial recruitment processes might have led to changes in behavior among patients assigned to the control treatment. Disappointment biases, including resentful demoralization, have been identified by previous studies of behavioral interventions.^33,34^ It is possible that some control patients might have felt uneasy or anxious if they perceived that telehealth had benefits that they were excluded from receiving for 12 months and this contributed to their decision to attend emergency rooms.* Participants can also react strongly to information regarding the likely effectiveness of new treatments, even if this is implied rather than explicit.^35^ The materials that were provided to patients by the WSD evaluation team were scrutinized by an independent research ethics committee to ensure that they were as neutral as possible, but some of the participating study sites nevertheless advertised and promoted telehealth in local media and through patient advocates.^36^

Pre–post analysis was not possible for mortality, so there is less direct evidence about the generalizability of this endpoint. However, the mortality effect estimated in WSD (odds ratio 0.54) is larger than other evaluative work in this area.^37^ A review of systematic reviews concluded that it is probable that mortality from heart failure can be reduced with telemonitoring,^38^ but evidence was less strong for diabetes and COPD, for which meta-analyses have tended to find no effect.^39–41^ The effect detected by WSD was across all 3 conditions but was larger than meta-analyses have found for heart failure alone. It is possible, but unlikely, that an artifact that led to the increases in emergency admissions could also explain the mortality effect. Admissions do have some associated risks, such as adverse events from invasive interventions or hospital-acquired infection.^42^

Concern about the generalizability of WSD, and thus the benefits of telehealth, appear to explain partly why the spread of telehealth has been limited.^43,44^ We will now show how the generalizability of treatment effects can be assessed empirically using placebo tests.

## Statistical Methods to Assess Generalizability

Like most RCTs, WSD reported the treatment effect for patients recruited into the trial, the sample average treatment effect (SATE).^11^ Equivalent estimands are the sample average treatment effect for the treated (SATT), which is conditional on assignment to the intervention arm, and likewise for controls (SATC).^†^ However, decision making usually requires an estimate of the treatment effect for the population that would be eligible for the treatment in routine practice, that is, the population average treatment effect (PATE), or for those who would receive the treatment in routine practice, that is, the population average treatment effect for the treated (PATT).

We define RCT results to be generalizable if it is possible to adjust sample average treatment effects to provide an unbiased estimate of the population effect. Hartman and colleagues^14,45^ specified conditions under which such adjustments can be unbiased:

- The treatments received in the trial are sufficiently consistent with those in routine practice so as to not have a differential effect on outcomes.
- There are no unobserved confounders in the selection of the RCT sample from the target population, analogous to the assumption of no unobserved confounding in observational studies.^46^

Hartman and colleagues tested these assumptions by comparing outcomes between the RCT and the target population for patients who received the same treatments, using equivalence-based placebo tests.^14,47,48^ These placebo experiments reverse the hypotheses of standard tests, so that the null hypothesis becomes that the groups have meaningfully different outcomes, while the alternative hypothesis is that the outcomes are similar. Reversing the hypotheses in this way avoids a problem of standard tests, in which lack of evidence for difference can be confused with evidence for similarity. In placebo tests, rejecting the null hypothesis provides support for the generalizability of the trial estimates, as both assumptions A and B will be valid (assuming contrasting effects did not cancel out). In contrast, failure of the placebo tests implies there is no robust evidence to support these assumptions and so, rather than directly reweighting the trial-based estimates,^6,12^ alternative approaches may be warranted (discussed subsequently). We next apply placebo tests to the WSD trial.

## Applying Placebo Tests to the WSD Trial

### Methods

We take the target population to be the wider set of adults in the WSD sites with COPD, diabetes, or heart failure, and we test whether RCT controls experienced similar outcomes to those predicted by patients in the target population who received usual care. Our method could be readily applied to compare telehealth patients across settings, but we focus on the control group because telehealth has not yet been widely diffused in England. Our definition of “meaningfully different” is 17.5%, as for the original RCT sample size calculation.^23^

**Figure 2:**
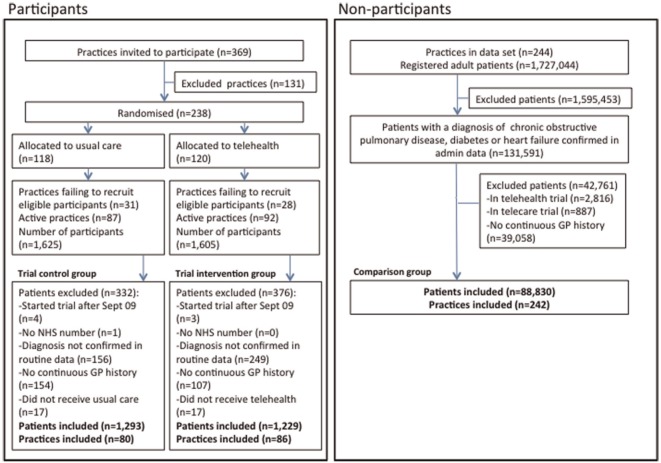
Flow diagram showing practice and patient recruitment. We excluded (from both participant and nonparticipant groups) people without a continuous record of registration with one or more primary care practices over the 2 years preceding the trial period. As per the original study, we excluded trial participants who were recruited after September 2009 or not linked to routine data. In the current study, we also excluded the small number of participants who did not receive their allocated treatments.

We identified the target population by applying standard diagnostic codes to the routine primary and secondary care data sets that were collected for the WSD evaluation (*n* = 88,830 eligible nonparticipants). To standardize inclusion criteria across our comparison groups, we excluded the relatively small number (*n* = 405) of RCT participants who had been identified by clinician reports, without a corresponding diagnosis in routine data. Figure 2 shows the flow of patients into the placebo tests. We identified many more eligible patients than the original WSD site teams (*n* = 91,647, including participants, compared with 15,171), but our estimate is more consistent with official estimates of disease prevalence (see Appendix B online) and highlights the need to assess the generalizability of the RCT.

We identified confounding variables by the following:

- Drawing on a qualitative study that explored the reasons given by patients for refusal to participate in the WSD trial^31^
- Reviewing existing observational studies of telehealth (see Appendix A)
- Considering factors predictive of emergency admissions^49^

We identified 65 baseline variables from the routine data, including the Combined Model score, which represented the estimated probability of emergency hospitalization during the trial period.^50^ Other baseline variables were related to demographics, physiological measurements (such as blood pressure), prescribed medications, diagnoses of health conditions, prior primary and secondary care use, area-level socioeconomic deprivation, and primary care practice characteristics (see Appendix B for the full list, Tables B1–B3).

We matched nonparticipants to RCT control patients using genetic matching, which is a computer-intensive search algorithm that can obtain more closely balanced groups than traditional methods such as pairwise matching on the propensity score.^8^ While baseline variables were generated for RCT control patients at their enrollment dates, nonparticipants were assigned multiple “index dates.” Specifically, each nonparticipant provided the matching algorithm with up to 14 observations corresponding to month-ends during the trial enrollment period (assuming the nonparticipant remained eligible for the placebo tests throughout). As any of these observations could be selected as the match for a particular RCT control patient, this approach increased our ability to find well-balanced groups. Genetic matching was applied separately to subgroups of patients defined by study site and chronic condition; within these subgroups, each RCT control patient was matched to 1 nonparticipant observation, with replacement. Balance was assessed within and across subgroups using the standardized difference, defined as the difference in sample means as a proportion of the pooled standard deviation.^51^ A threshold of 10% has been used to describe meaningful imbalances.^52^ We also compared distributions of baseline variables between groups using the variance ratio and quantile-quantile plots.^53^

After matched patients had been selected, we calculated utilization and mortality outcomes over 12 months,^‡^ as in the primary studies.^24,27^ Our main analysis compared each outcome between RCT controls and matched nonparticipants using generalized linear models, adjusting for residual imbalances in baseline covariates to estimate SATC. The generalized linear models were assumed to follow a Poisson distribution with log link for the utilization outcomes. Robust standard errors were used to reflect the estimated covariance structure of the data, including the clustering of patients within general practices.

Results from the Poisson regression were presented as incidence rate ratios for RCT controls compared with matched nonparticipants. The result of each placebo test was reported according to the confidence interval obtained for the rate ratio. For example, if the point estimate and 95% confidence interval were bounded within a range that corresponded to a 17.5% difference (i.e., within 0.825–1.175), the placebo test passed, and we concluded that there was evidence that the RCT control and nonparticipant groups had similar values of this endpoint. Otherwise, the placebo test failed. For mortality, models used logistic regression rather than Poisson regression and the placebo test was according to the estimated odds ratio.

Matching prior to regression should reduce the sensitivity of the results to the specification of the regression model,^54^ but we also undertook additional robustness checks. These included specifying time-series models to exploit the longitudinal nature of the routine data sets (see Appendix B).

### Results

**Figure 3:**
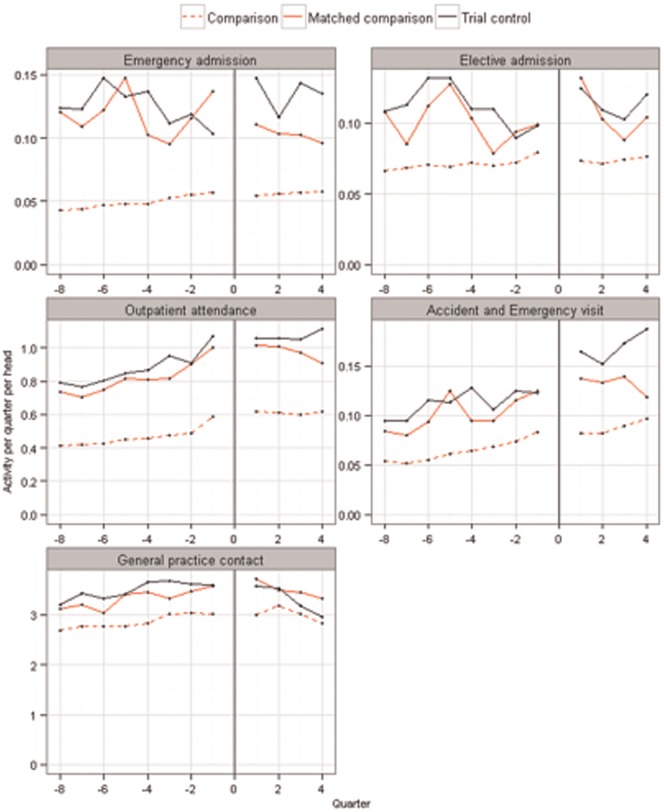
Crude trends in rates of service use (contacts per patient per quarter). The observations to the left of the vertical line show rates of primary and secondary care contact for each of the 8 calendar quarters preceding trial recruitment (i.e., for a total of 2 years). The observations to the right of the vertical line show rates for survivors for the 4 quarters in the trial period. A gap has been imposed at the time of recruitment for clarity. Rates for RCT control patients (*n* = 1293) are shown in black. The dashed red line shows rates for the eligible nonparticipants (*n* = 88,830), while the solid red line shows rates for the matched subgroup of eligible nonparticipants (*n* = 1293). These were matched to RCT controls on variables including prior rates of primary and secondary care contact, and the placebo tests assess whether rates continued to be similar during the trial period. For the purposes of producing this figure, comparison patients were randomly allocated index dates in approximately the same distribution as trial control patients.

Before matching, nonparticipants had less severe case-mix than RCT controls (mean Combined Model score 0.16 v. 0.26, standardized difference 57.2%). After matching, both groups had mean Combined Model score equal to 0.26 (standardized difference 0.3%). Of the 65 baseline variables, only 3 had standardized differences that were above the 10% threshold after matching, namely rates of never-smokers, atrial fibrillation, and COPD, as recorded in primary care data (standardized differences 11.4%, 10.5% and 10.3%, respectively).^#^ See Table 1 for a summary and Appendix B for detail (Tables B1–B3). The groups also had similar historic trends in service use (Figure 3).

**Table 1:** Selected Baseline Variables

	Nonparticipants (*n* = 88,830)	Trial Control Group (*n* = 1,293)	Matched Nonparticipants for Trial Control Group (*n* = 1,293)	Standardized Difference (Variance Ratio)	
				Before Matching	After Matching
Practice list size, number of patients per practice	9088 (4814)	10,041 (5944)	10,071 (5758)	17.6 (1.52)	−0.5 (1.07)
Age in years	66.4 (14.3)	70.8 (11.3)	70.8 (11.1)	34.0 (0.63)	−0.5 (1.04)
Female, %	46.2	40.3	41.1	−11.9	−1.6
Chronic obstructive pulmonary disease, %	24.7	60.0	60.2	76.4	−0.3
Diabetes, %	70.7	34.7	35.5	−77.4	−1.6
Heart failure, %	12.9	35.2	36.7	54.1	−3.1
Number of chronic conditions per head	0.90 (1.34)	1.76 (1.80)	1.73 (1.77)	54.2 (1.80)	2.1 (1.03)
Combined model score	0.16 (0.15)	0.26 (0.20)	0.26 (0.20)	57.2 (1.78)	0.3 (1.00)
Had 10 or more medicines prescribed, %	7.3	15.9	16.0	27.2	−0.2
Hemoglobin A1c^a^	7.37 (1.63)	8.38 (1.74)	8.23 (1.64)	60.0 (1.13)	8.6 (1.13)
Current smoker, %	17.6	20.0	19.1	6.0	2.1
Service use per head, 1–360 days before index date					
Emergency admissions	0.21 (0.67)	0.47 (1.07)	0.45 (0.94)	29.0 (2.60)	2.1 (1.30)
Elective admissions	0.29 (0.99)	0.41 (1.03)	0.38 (0.94)	11.2 (1.07)	3.3 (1.19)
Emergency room visits	0.29 (0.88)	0.48 (1.12)	0.43 (0.94)	19.0 (1.63)	5.1 (1.43)
Outpatient attendances	2.01 (4.01)	3.80 (5.44)	3.53 (4.91)	37.4 (1.84)	5.2 (1.22)
Primary care contacts	11.92 (12.06)	14.55 (12.13)	13.85 (11.00)	21.8 (1.01)	6.1 (1.21)

Note: Data show mean and standard deviation unless otherwise stated. For information on the full set of 65 baseline variables, see Appendix B.

a. For the diabetes subset only (*n* = 272 intervention patients; 272 matched controls).

During the trial, RCT controls experienced similar rates of outpatient attendance, primary care contact, and elective admission as matched nonparticipants (Table 2). The placebo tests passed for outpatient attendances and primary care contacts (see Table 3, column A). For example, the rate ratio for outpatient attendances was 1.03 (95% CI 0.94–1.13). In contrast, RCT controls experienced more emergency admissions than matched nonparticipants (rate ratio 1.22, 95% CI 1.05–1.43). They also had higher mortality by 12 months, albeit from a low base (5.7% v. 2.9%; odds ratio 2.17, 95% CI 1.16–4.08). The placebo tests therefore failed for emergency admissions and mortality.

**Table 2:** Utilization and Mortality Endpoints during the 12 Months following the Index Dates

	Trial Control Group (*n* = 1293)	Matched Nonparticipants for Trial Control Group (*n* = 1293)	Trial Intervention Group (*n* = 1229)	Matched Nonparticipants for Trial Intervention Group (*n* = 1229)
Emergency admissions per head	0.54 (1.24)	0.41 (0.93)	0.46 (1.02)	0.40 (0.88)
Elective admissions per head	0.46 (1.24)	0.43 (1.09)	0.41 (0.98)	0.50 (2.68)
Outpatient attendances per head	4.28 (6.19)	3.90 (5.20)	4.46 (6.03)	4.05 (5.35)
Emergency room visits per head	0.68 (1.51)	0.53 (1.26)	0.58 (1.21)	0.58 (1.26)
Primary care contacts per head	13.24 (13.08)	14.00 (12.43)	13.57 (12.33)	12.87 (10.79)
Mortality, percentage of patients	5.7 (*n* = 74)	2.9 (*n* = 37)	2.8 (*n* = 34)	2.1 (*n* = 26)

Note: Data show mean and standard deviation unless otherwise stated.

**Table 3:** Results of the Generalized Linear Models

	Placebo Tests	Estimated Effect of Telehealth	
	(A) Comparison of Trial Control Group v. Corresponding Matched Nonparticipants	(B) RCT Estimate (Compares Trial Intervention Group with Trial Controls)	(C) Sensitivity Analysis (Compares Trial Intervention Group with Corresponding Matched Nonparticipants)
Emergency admissions per head	1.22 (1.05–1.43)	0.90 (0.77–1.05)	1.12 (0.95–1.31)
Elective admissions per head	0.99 (0.83–1.18)	0.95 (0.80–1.14)	0.87 (0.73–1.05)
Outpatient attendances per head	1.03 (0.94–1.13)^a^	1.02 (0.93–1.12)	1.04 (0.95–1.14)
Emergency room visits per head	1.23 (1.07–1.43)	0.86 (0.74–0.99)	0.96 (0.83–1.11)
Primary care contacts per head	0.92 (0.87–0.97)^a^	1.06 (1.01–1.13)	1.04 (0.99–1.09)
Mortality	2.17 (1.16–4.08)	0.41 (0.13–1.23)	1.50 (0.57–3.94)

Note: Estimates are for the incidence rate ratio for the trial controls v. comparison populations and accompanying 95% confidence intervals, except for mortality, where odds ratios are reported.

a. These placebo tests pass, as the point estimate and 95% confidence interval are contained within the range (0.825–1.175).

### Interpretation of the Placebo Tests

One explanation for the failure of the placebo tests for emergency admissions and mortality is that the RCT data did not include all confounders of sample selection for these endpoints. Compared with other telehealth studies (see Appendix A), we were in a relatively strong position to deal with confounding, with 65 baseline variables. We were able to identify retrospectively a group of nonparticipants who met the inclusion criteria for the WSD trial, and we also had access to a qualitative study of reasons for nonparticipation in the trial.^31^ As the electronic medical record was available, we were not confined to nonclinical, administrative data.^55^ Genetic matching produced good balance, both overall and within subgroups defined by study site and chronic condition, thus avoiding confounding through recognizing some important interaction effects. Furthermore, our definition of the target population meant that we selected nonparticipants from within the same geographical areas as RCT participants. This was expected to reduce confounding due to area-level effects (such as those associated with healthcare providers),^56^ although we also matched on the characteristics of primary care practices. Additional analysis (see Appendix B) provided reassurance that the placebo tests were not sensitive to the specification of the regression models.

Any remaining confounding variables would need to be unobserved (as good balance was obtained on observed variables) and not strongly predictive of primary care contacts, outpatient attendances, and elective admissions (as no differences were seen on those variables). Some candidates are general attitudes toward using emergency care and severity of illness requiring emergency care. These, however, are correlated with variables for which we did control, such as prior emergency admissions,^57^ and it is not obvious how they would lead to the sudden increases in admissions that were observed among the RCT control group.

A plausible explanation for the failure of the placebo tests for emergency admissions is that the implementation of the trial protocol altered unplanned forms of care for RCT patients assigned to receive usual care. As the placebo tests reported similar rates of elective admission, outpatient attendance, and primary care contact (all of which are planned by an appointments system), it now seems unlikely that the healthcare professionals involved in recruiting patients for the RCT altered the management of patients assigned to the control. However, it is possible that the trial recruitment processes led to changes in behavior amongst these patients. For example, recruitment into the trial could have increased unease or anxiety among those with a preference for telehealth, such that patients were more likely to seek help at emergency rooms.^58^ Unfortunately, information on patient preferences was not collected during the trial,^21^ so it is not possible to verify this theory directly.

Compared with emergency admissions, confounding appears more likely for mortality. In-hospital mortality is strongly predicted by clinical information recorded at the point of admission, such as blood pressure and respiratory rate,^59,60^ but this information was not available within our data sets at that particular point in the care pathway. It is hard to see how the implementation of the trial protocol could have altered usual care to the extent that mortality rates were significantly increased, although it is true that the mortality effect estimated from the RCT data was unusually large.

Regardless of the explanation for the failure of the placebo tests for emergency admissions and mortality, the conclusion is the same: The generalizability of the WSD trial was limited for these endpoints. This encourages sensitivity analyses to determine whether the trial-based estimates are robust to alternative comparison groups.

## Sensitivity Analysis Regarding Reasons for Nongeneralizability

### Methods

Failure of the placebo tests implies that the control treatment in the trial was different than that received in the target population or the sample selection was confounded (conditional on observables), or both. Although analysis of RCT data will give unbiased estimates of sample treatment effects, in applying these findings it is necessary to assume that the treatments are consistent with those in routine practice. We can produce an alternative estimate of the sample treatment effect by comparing the RCT intervention patients with a comparable group of patients receiving the control treatment within the target population. This comparison is observational (and thus susceptible to confounding), but it avoids having to assume that the RCT control group experienced care consistent with usual care in routine practice. We propose that reporting sample treatment effects using both the RCT controls (base case) and a matched sample from the target population (sensitivity analysis) offers a useful check on whether the results from the RCT are robust to these threats to generalizability.**

To apply the sensitivity analysis to the WSD study, we matched nonparticipants to patients in the RCT telehealth arm, using the same approach as before. After satisfactory balance had been obtained (see Appendix C), we applied generalized linear models to the matched data to estimate the SATT of telehealth versus usual care, by contrasting endpoints for the RCT telehealth arm with those of the matched nonparticipant patients who received usual care. We also obtained the comparable estimate from the RCT, by matching controls and telehealth patients within the trial and fitting generalized linear models.

### Results

For those endpoints where the placebo tests passed, estimated treatment effects were similar regardless of whether telehealth patients were compared with RCT controls (see Table 3, column B), or with matched nonparticipants (column C). Thus, for example, both analyses found that telehealth did not significantly change the use of outpatient services, and both analyses reported similar point estimates for primary care contacts (although significance levels varied slightly).^††^ In contrast, the estimated treatment effects for emergency admissions and mortality differed markedly according to the comparison group. Thus, although analysis of RCT data alone suggested reductions in emergency admissions (rate ratio 0.90, 95% CI 0.77–1.05),^‡‡^ the comparison between telehealth and matched nonparticipants reported a trend toward more emergency admissions among telehealth patients (rate ratio 1.12, 95% CI 0.95–1.31). Likewise, analysis of RCT data alone suggested that telehealth reduced mortality (odds ratio 0.41, 95% CI 0.13–1.23), but the comparison with nonparticipants reported a trend in the opposite direction (odds ratio 1.50, 95% CI 0.57–3.94). These sensitivity analyses have implications for policy making because reductions in emergency admissions continue to be a major motivation to invest in telehealth.

## Discussion

The current paper adds to a growing body of research addressing the potential of observational data to assess and strengthen the generalizability of RCT-based estimates of comparative effectiveness.^6,12,14^ Hartman and colleagues^14^ proposed placebo tests for assessing the assumptions required for RCTs to provide unbiased estimates of population average treatment effects. We applied placebo tests to an RCT of a complex out-of-hospital intervention, comparing the outcomes of RCT control patients with those of matched nonparticipants receiving usual care. Unlike previous studies,^6,12,14^ we proposed sensitivity analyses to explore the implications of alternative assumptions about the cause of nongeneralizability. Sensitivity analyses may be particularly useful for decision makers in the settings exemplified by our case study, where placebo tests indicate that the main trial findings do not generalize to the target population of interest. For WSD, they showed that if one assumes that the placebo tests failed because of differences in control treatments between settings, then the data were consistent with an increase in emergency hospital admissions due to telehealth.

Our approach to placebo tests and associated sensitivity analyses has the following requirements. First, before conducting the placebo tests, the target population must be defined, along with what constitutes a meaningful difference in outcomes. In our example, the target population consisted of local patients with the relevant chronic conditions, and the definition of “clinically different” came from the original sample size calculation.

Second, RCT investigators should understand reasons for nonparticipation in an RCT. Just as in an observational study, it is vital to understand the mechanism for treatment selection.^61^ In our example, we drew on a qualitative study about reasons for refusal to participate in the WSD trial.^31^

Third, observational data sets are required with patients, contexts, and treatments that represent the target population and overlap with those included in the RCT. These data sets must contain sufficient information to reproduce the main RCT eligibility criteria and outcomes. One possibility is to collect baseline, process, and follow-up data for people who refused to participate in the RCT, as in a comprehensive cohort study.^62,63^ Another is to embed RCTs within large routine data sets.^64^

Fourth, placebo tests should use analytical techniques that are able to address confounding. We used genetic matching,^8^ since the large number of baseline variables (*n* = 65) was too challenging for traditional matching methods. We also carefully assessed the sensitivity of the placebo tests to alternative regression model specifications (see Appendix B), including the addition of interaction terms and alternative model structures.

Fifth, as in our example, the study is required to prespecify sensitivity analysis to assess the robustness of the findings.

Our study has a number of limitations. The placebo tests addressed only some aspects of generalizability, related to the characteristics and treatments of the control group. The generalizability of the intervention regimen could have been addressed with very similar methods, but data were not available on patients receiving telehealth in routine settings. Also, although the placebo tests are useful for providing a quantitative assessment of the generalizability of the RCT-based estimates, they cannot identify precisely why a study was not generalizable. We believe that the most plausible reason for the failure of the placebo tests for emergency admissions in the WSD study was that the control patients received atypical care or otherwise reacted to their treatment allocations, but it is also possible that there were important unmeasured differences in patient characteristics between settings. Finally, as with many comparative effectiveness studies, several endpoints were measured with no attempt to allow for multiplicity. Where the placebo tests indicate that treatment effects were more generalizable for some endpoints than others, judgment is required to decide whether to proceed to estimating population average treatment effects. One explicit approach to weighting the alternative endpoints would be to apply the placebo tests to overall metrics such as those used in cost-effectiveness analyses (e.g., to contrast net monetary benefits between the RCT and routine practice settings).

Future research could address alternative estimators for the placebo tests. Stuart and colleagues^12^ used 3 methods to reweight control outcomes to the target population, namely inverse probability of treatment weighting, full matching, and subclassification. These gave similar results in their example. We found that generalized linear modeling and time series regression gave similar results (see Appendix B), but further investigation is warranted. Placebo tests could also be extended to consider missing endpoints data and could be considered for situations without such a rich set of baseline variables as WSD.

This study raises the question about the value of collecting observational data alongside RCTs, given that the amount of funding for a given RCT is constrained and there are competing priorities. Substantial efforts are often made at the design stage to improve the generalizability of RCTs,^2^ but analytical-stage strategies that assess generalizability empirically are often relatively limited. The CONSORT statement, for example, recommends only a table of baseline characteristics.^65^ We conclude that the proposed placebo tests and accompanying sensitivity analyses provide information about the level of generalizability actually achieved in RCTs. These additions to the methodological toolkit can help decision makers judge the extent to which estimates of comparative effectiveness obtained from RCTs can be adjusted to apply to their target populations.

## Supplementary Material

Supplementary material

## Supplementary Material

Supplementary material

## Supplementary Material

Supplementary material

## Supplementary Material

Supplementary material

## Supplementary Material

Supplementary material

## Supplementary Material

Supplementary material

## Supplementary Material

Supplementary material

## Supplementary Material

Supplementary material

## Supplementary Material

Supplementary material

## Supplementary Material

Supplementary material

## Supplementary Material

Supplementary material

## Supplementary Material

Supplementary material

## Supplementary Material

Supplementary material
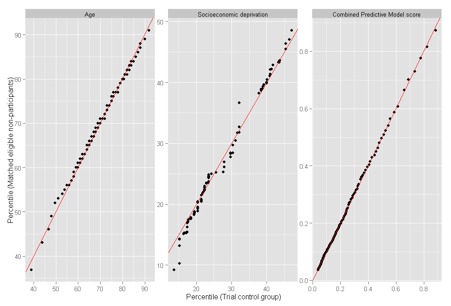

## Supplementary Material

Supplementary material
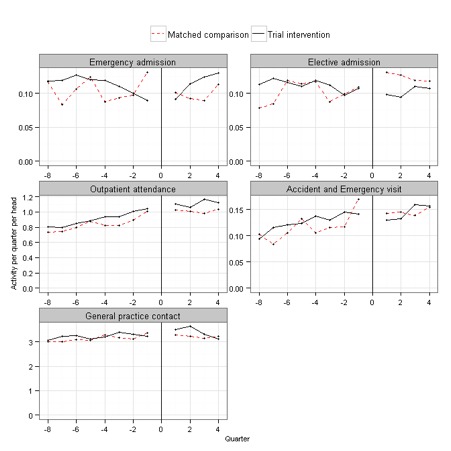

## Footnotes

* A study that was nested within the WSD trial reported higher anxiety among controls than telehealth patients 4 months after recruitment, but these differences did not reach statistical significance.^66^ As this study did not have information on nonparticipants, it was not possible to test whether this difference was an effect of telehealth or an artifact of the trial.

† These estimands are asymptotically equivalent because randomization implies that potential outcomes in the treatment and control groups are exchangeable. They will in general not be equivalent in nonrandomized settings.

‡ One minor difference is that, while the original evaluation used national hospital data from the Hospital Episode Statistics (HES), we used the local equivalent (the Secondary Uses Service, or SUS). SUS enabled us to link anonymized hospital and primary care data across the local populations and not just across WSD participants. Because SUS is the source data for HES, levels of agreement were very high. However, unlike HES, our SUS data would not continue to track hospital activity if a resident of a WSD area moved away from that area.

# Atrial fibrillation and COPD showed more similar prevalence on hospital data (standardized differences of 7.4% and 1.4%, respectively), suggesting greater balance was achieved for a subset of patients of more severe case mix than overall. The groups reported similar rates of current smokers and ex-smokers (standardized differences of 2.1% and 8.7%, respectively).

** The proposed sensitivity analysis has some similarities with previous comparisons between the outcomes of people receiving new treatments in RCTs versus those of people receiving control treatments in routine practice.^67^ However, these have often been concerned with testing the ability of the analytical methods to adjust for selection bias. We argue that because the selection mechanism involved relates to recruitment into the RCT, the current comparisons are fundamentally related to the generalizability of the RCT.

†† The original WSD evaluation found no statistically significant differences in rates of primary care contact.^27^ However, unlike the current study, the original evaluation examined 2 types of primary care contact separately (those with general practitioners and those with practice nurses). Estimands also differed, and the matching algorithm adopted in the current study may have increased the precision of its estimator.

‡‡ The original analysis of the RCT data reported statistically significant associations between telehealth and emergency admissions, and likewise for mortality.^24^ The analysis of RCT data in Table 3 reported nonsignificant effects for both, but the sample size was smaller.
