# Supplementary material for: An Approach to Assess Generalizability in Comparative Effectiveness Research: A Case Study of the Whole Systems Demonstrator Cluster Randomized Trial Comparing Telehealth with Usual Care for Patients with Chronic Health Conditions
Source: Med Decis Making. 2015 Nov;35(8):1023–36. doi: 10.1177/0272989X15585131 (PMC4592957; doi:10.1177/0272989X15585131)
Supplement: Supplementary material [file DS_10.11770272989X15585131_Appendix.pdf]

DOI: 10.1177/0272989X15585131

Web-only appendix A: Confounders identified in previous matched control studies of telehealth

DOI: 10.1177/0272989X15585131

References

1. Pekmezaris R, Mitzner I, Pecinka KR, Nouryan CN, Lesser ML, Siegel M, et al. The impact of remote patient monitoring (telehealth) upon Medicare beneficiaries with heart failure. Telemed J E Health. 2012 Mar;18(2):101–8.

2. Sohn S, Helms TM, Pelleter JT, Müller A, Kröttinger AI, Schöffski O. Costs and benefits of personalized healthcare for patients with chronic heart failure in the care and education program "Telemedicine for the Heart". Telemed J E Health. 2012 Apr;18(3):198–204.

3. Morguet AJ, Kühnelt P, Kallel A, Jaster M, Schultheiss H-P. Impact of telemedical care and monitoring on morbidity in mild to moderate chronic heart failure. Cardiology. 2008 Jan;111(2):134–9.

4. Barnett TE, Chumbler NR, Vogel WB, Beyth RJ, Qin H, Kobb R. The effectiveness of a care coordination home telehealth program for veterans with diabetes mellitus: a 2-year follow-up. Am J Manag Care. 2006 Aug;12(8):467–74.

5. Jia H, Chuang H-C, Wu SS, Wang X, Chumbler NR. Long-term effect of home telehealth services on preventable hospitalization use. J Rehabil Res Dev. 2009 Jan;46(5):557–66.

6. Jia H, Feng H, Wang X, Wu SS, Chumbler N. A longitudinal study of health service utilization for diabetes patients in a care coordination home-telehealth programme. J Telemed Telecare. 2011 Jan;17(3):123–6.

7. Chumbler NR, Chuang H-C, Wu SS, Wang X, Kobb R, Haggstrom D, et al. Mortality risk for diabetes patients in a care coordination, home-telehealth programme. J Telemed Telecare. 2009 Jan;15(2):98–101.

8. Sicotte C, Paré G, Morin S, Potvin J, Moreault M-P. Effects of home telemonitoring to support improved care for chronic obstructive pulmonary diseases. Telemed J E Health. 2011 Mar;17(2):95–103.

9. Nilsson M, Rasmark U, Nordgren H, Hallberg P, Skönevik J, Westman G, et al. The physician at a distance: the use of videoconferencing in the treatment of patients with hypertension. J Telemed Telecare. 2009 Jan;15(8):397–403.

10. Baker LC, Johnson SJ, Macaulay D, Birnbaum H. Integrated telehealth and care management program for Medicare beneficiaries with chronic disease linked to savings. Health Aff (Millwood). 2011 Sep;30(9):1689–97.

11. Chen H-F, Kalish MC, Pagan JA. Telehealth and hospitalizations for Medicare home healthcare patients. Am J Manag Care. 2011 Jun;17(6 Spec No.):e224–30.

Web-only appendix B: Matching and placebo tests

Numbers of eligible patients

The WSD site teams identified 15,171 potentially eligible patients using a search of routine primary and secondary care data, whereas we applied standard diagnostic codes to similar data sets and found a much higher number of eligible patients (88,830 non-participants plus 2,817 of participants, i.e., 91,647 in total). Table B2 shows that 24.7% of the non-participants we identified had chronic obstructive pulmonary disease, while 70.7% had diabetes and 12.9% had heart failure.

We sense checked our figures by comparing them with estimates from the system of performance-related pay that exists for general practices in England, the Quality Outcomes Framework (QOF). QOF estimates are based using routine primary care data only and, for diabetes, relate to the population aged 17 or over rather than 18 or over. For 2009/10, QOF reported 35,360, 104,560, and 15,903 people with chronic obstructive pulmonary disease, diabetes, and heart failure, respectively in the three WSD sites. Some people will have several of these conditions.

We would expect the QOF figures to be higher than ours because they are based on all diagnostic codes recorded on the primary care data, however long ago these were recorded. Our primary care data sets, although very extensive with over one billion records, were generally restricted to the period April 2006 to September 2010 (*i.e.*, they started around 3.5 years before recruitment began in September 2009).

Based on the comparison with QOF, we believe that our estimates of the number of eligible patients (n=91,647) are plausible. Recruitment into the WSD trial was a complex and time-consuming process that required formal patient consent to be given twice. Various teams had to

be carefully coordinated, including the WSD site team, general practices, patients, telehealth installation experts, a market research company (for baseline questionnaires), and social care (for a linked trial of telecare). Therefore, recruitment of patients happened successively for batches of patients, until the target number had been reached (n=3,000 across the three sites).

Matching

Baseline variables were calculated as at the trial start date for trial patients and, for non-participants, at up to 14 "index dates" spanning the trial recruitment period (month ends from July 2008 to August 2009). We excluded potential comparison patients who had died before a given index date or whose diagnosis of a chronic condition did not occur before this date.

Matching was performed separately for strata defined by chronic condition and site (*i.e.,* 9 strata in total). Within each stratum, one matched comparison patient was selected for each trial patient with replacement, using Genetic Matching.(1) This is a computer-intensive approach that searches over a space of distance measures. In this context, a distance measure evaluates the similarity of two patients at baseline, as a function of their baseline variables. Various measures are possible, with different weights attached to the constituent variables. For each sampled measure, the genetic matching algorithm assembles matched pairs and assesses balance on baseline variables. Thus, the algorithm attempts to find the distance measure that gives the optimal level of balance. Balance was assessed by p-values from paired t-tests as well as, for continuous variables, p-values from Kolmogorov-Smirnov tests. The genetic matching algorithm was run with a population size of 2,000, and stopped after no improvement was detected within 100 generations.

Matched comparison patients selected across the strata were then recombined for the analyses. Means and variances are reported in Tables B1-B3, while quantile-quantile plots of the three continuous variables are shown in Figure B1. Most baseline variables had no missing data. For

DOI: 10.1177/0272989X15585131

baseline variables for which there was some missing data (such as blood pressure readings), balance statistics for that variable are reported for complete cases.

Generalized linear regression

After matched data sets had been assembled, analytical models were applied. Regression models were constructed using generalized linear modeling with a log link, and robust standard errors. The headline results presented in the paper are adjusted for a set of 51 baseline variables; however, we tested a range of other model specifications to assess the robustness of the results. The parsimonious model adjusted for 25 variables selected manually by removing and adding variables in such a way as to optimize model fit according to the Akaike Information Criterion (AIC). More extensive models included interaction terms with age, site and indexed chronic condition, again selected with reference to the AIC. Results for the estimated difference in emergency hospital admissions were robust to changes in model specification on the matched data sets, with only small changes detected in point estimates and confidence intervals (Table B4).

Time series analysis

In addition to the generalized linear models, we also considered addressing residual confounding after matching by applying repeated measures ('time series') models to the matched data. These time series models used quarterly utilization totals spanning the period from two years before the date of enrolment into the trial (or the index date, for non-participants) to 12 months afterwards, and included an interaction term between group membership and trial period. Error terms were assumed to be autoregressive with order one;(2) robust standard errors were applied. Compared with the generalized linear models, the time series models were expected to reduce the risk of unobserved confounding, provided that the unobserved confounder did not have an effect that varied with time. However, it was not possible to apply time series models to the mortality data,

as no deaths occurred in the study cohorts before the index dates. Results from the time series models were similar to those from generalized linear modeling (Table B5). Effect sizes from the time series models were also robust to alternative model specification (Table B6).

References

(1) Sekhon JS, Grieve RD. A matching method for improving covariate balance in cost-effectiveness analyses. Health Econ. 2012 Jun;21(6):695–714.

(2) Cowpertwait PSP, Metcalfe A V. Introductory Time Series with R. Dordrecht: Springer; 2009.

DOI: 10.1177/0272989X15585131

Web-only appendix C: Analysis for trial intervention group

Tables C1-C3 show the results of the matching algorithm when applied to the trial intervention group. Figure C1 shows trends in utilization for the trial intervention group and the corresponding matched eligible non-participants. Table C4 shows estimated treatment effects using both generalized linear modeling and the alternative modeling approach based on time series analysis (see Appendix B for a description of the time series modeling).

Before matching, non-participants had less severe case-mix than RCT intervention patients (mean Combined Model score 0.16 *vs.* 0.26, standardized difference 57.4%). After matching, both groups had mean Combined Model score equal to 0.26 (standardized difference 0.9%). Out of the 65 baseline variables, six had standardized differences above the 10% threshold after matching, namely diastolic blood pressure and rates of current smoking, ex-smoking, atrial fibrillation, dementia, and mental health conditions recorded on primary care data (10.5%, -12.1%, 18.0%, 11.9%, -14.4% and -16.4%). The groups also had similar historic trends in service use (Figure C1).
