# Supplementary material for: An Approach to Assess Generalizability in Comparative Effectiveness Research: A Case Study of the Whole Systems Demonstrator Cluster Randomized Trial Comparing Telehealth with Usual Care for Patients with Chronic Health Conditions
Source: Med Decis Making. 2015 Nov;35(8):1023–36. doi: 10.1177/0272989X15585131 (PMC4592957; doi:10.1177/0272989X15585131)
Supplement: Supplementary material [file DS_10.11770272989X15585131_TableC3.pdf]

DOI: 10.1177/0272989X15585131

Table C3: Balance, before and after matching, in the sensitivity analysis as applied to the RCT intervention group (person-level variables, continued)

	Non-participants (n=88,830)	Trial intervention patients (n=1,229)	Matched non-participants (n=1,293)	Standardised difference (variance ratio)	
				Before matching	After matching
**Health conditions recorded on hospital data**					
COPD	8.0	26.4	23.0	50.1	7.7
Congestive heart failure	5.7	15.1	15.4	31.2	-0.7
Diabetes	24.2	27.2	28.2	6.9	-2.2
Cancer	4.9	6.8	6.5	7.9	1.0
Cancer (benign)	1.4	2.2	1.9	5.9	2.3
Alcohol abuse	0.8	1.4	1.1	5.6	3.0
Hypertension	22.7	37.7	38.5	33.0	-1.7
Injury	8.3	13.8	12.9	17.3	2.6
Iatrogenic	2.9	5.7	5.2	13.9	2.2
Falls	3.4	5.2	5.1	8.9	0.4
Mental health	3.6	4.9	6.0	6.4	-5.0
Angina	5.7	13.1	12.6	25.5	1.5
Ischemic heart disease	10.9	25.1	24.2	37.9	2.1
Asthma	5.8	12.6	12.9	23.8	-0.7
Anaemia	3.5	6.4	6.6	13.7	-0.7
Atrial fibrillation	6.9	16.4	13.9	29.7	6.8
Cerebrovascular disease	3.3	6.0	5.9	12.8	0.7
Peripheral vascular disease	3.1	7.1	6.0	18.4	4.3
Renal failure	2.7	5.6	3.9	14.6	8.0
Respiratory infection	2.8	7.9	6.3	22.6	6.0
Number of chronic conditions per head (mean (SD))	0.90 (1.34)	1.72 (1.78)	1.67 (1.73)	52.1 (1.75)	3.0 (1.06)
**Health conditions recorded on primary care data**					
Atrial fibrillation	8.4	17.9	13.6	28.3	11.9
Cancer	7.2	9.4	9.2	8.0	0.6
Coronary heart disease	19.7	35.0	34.3	34.9	1.4
Chronic kidney disease	17.6	22.9	22.6	13.2	0.6
COPD	22.0	51.8	47.6	65.0	8.5
Dementia	1.5	0.3	1.8	-12.0	-14.4
Depression	14.8	17.8	19.9	8.2	-5.4
Diabetes	67.8	43.4	44.3	-50.6	-1.6
Heart failure	10.0	29.9	27.8	51.4	4.5
Hypertension	52.7	52.7	54.2	0.0	-2.9
Mental health	1.6	0.2	2.0	-14.1	-16.4
Asthma	13.8	19.8	19.0	16.0	2.1

DOI: 10.1177/0272989X15585131

Stroke / TIA	7.1	9.5	9.4	8.8	0.3

Note: TIA = Transient ischemic attack.
