# Supplementary material for: An Approach to Assess Generalizability in Comparative Effectiveness Research: A Case Study of the Whole Systems Demonstrator Cluster Randomized Trial Comparing Telehealth with Usual Care for Patients with Chronic Health Conditions
Source: Med Decis Making. 2015 Nov;35(8):1023–36. doi: 10.1177/0272989X15585131 (PMC4592957; doi:10.1177/0272989X15585131)
Supplement: Supplementary material [file DS_10.11770272989X15585131_TableC4.pdf]

Table C4: Estimates of the sample average treatment effect for the telehealth patients (figures are for the incidence rate ratio for the trial intervention group, except for mortality, where figures are for the odds ratio)

	Generalized linear modeling		Time series	
	Comparison with RCT control group	Comparison with non-participants	Comparison with RCT control group	Comparison with non-participants
Emergency admissions per head	0.90<br>(0.77, 1.05)	1.12<br>(0.95, 1.31)	0.77<br>(0.66, 0.91)	1.10<br>(0.92, 1.31)
Elective admissions per head	0.95<br>(0.80, 1.14)	0.87<br>(0.73, 1.05)	0.72<br>(0.58, 0.90)	0.75<br>(0.58, 0.98)
Outpatient attendances per head	1.02<br>(0.93, 1.12)	1.04<br>(0.95, 1.14)	1.00<br>(0.92, 1.10)	1.02<br>(0.93, 1.12)
Emergency room visits per head	0.86<br>(0.74, 0.99)	0.96<br>(0.83, 1.11)	0.66<br>(0.56, 0.78)	0.95<br>(0.80, 1.13)
Primary care contacts per head	1.06<br>(1.01, 1.13)	1.04<br>(0.99, 1.09)	1.09<br>(1.04, 1.16)	1.06<br>(1.01, 1.11)
Mortality	0.41<br>(0.13, 1.23)	1.50<br>(0.57, 3.94)	n/a	n/a

Note: Time series models were not defined for mortality, as no deaths occurred before the date of enrolment into the trial.
