# Supplementary material for: An Approach to Assess Generalizability in Comparative Effectiveness Research: A Case Study of the Whole Systems Demonstrator Cluster Randomized Trial Comparing Telehealth with Usual Care for Patients with Chronic Health Conditions
Source: Med Decis Making. 2015 Nov;35(8):1023–36. doi: 10.1177/0272989X15585131 (PMC4592957; doi:10.1177/0272989X15585131)
Supplement: Supplementary material [file DS_10.11770272989X15585131_TableA1.pdf]

DOI: 10.1177/0272989X15585131

Table A1: Confounders identified in other matched comparison studies of telehealth (remote patient monitoring)

First author and year	Conditions	Primary outcome	Demographics						Clinical status				Service use		Other	
			Age	Sex	Ethnicity	Marital status	Living alone	Socioeconomic score	Principal condition	Secondary conditions	Clinical disease severity	Medication	Hospital use	Primary care use	Patient priority	Predictive risk score
Pekmezaris 2012 (1)	Heart failure	Admissions	Y		Y				Y		Y		Y			
Sohn 2012 (2)	Heart failure	Costs	Y	Y					Y	Y	Y		Y			
Morguet 2008 (3)	Heart failure	Hospital length of stay	Y	Y					Y	Y	Y	Y				
Barnett 2006 (4)	Diabetes	Admissions	Y		Y	Y			Y	Y			Y			
Jia 2009 (5-7)*	Diabetes	Various	Y	Y	Y	Y			Y	Y			Y		Y	
Sicotte 2011 (8)	COPD	Various	Y	Y							Y					
Nilsson 2009 (9)	Hyper-tension	Blood pressure	Y	Y					Y							
Baker 2011 (10)	Various	Healthcare spending	Y	Y					Y	Y			Y	Y		Y
Chen 2011 (11)	Aged over 65	Admissions	Y	Y	Y		Y		Y	Y			Y			
Current study	Mixed	Admissions	Y	Y				Y*	Y	Y	Y**	Y	Y	Y		Y

* at general practice level ** for diabetes
