# Supplementary material for: An Approach to Assess Generalizability in Comparative Effectiveness Research: A Case Study of the Whole Systems Demonstrator Cluster Randomized Trial Comparing Telehealth with Usual Care for Patients with Chronic Health Conditions
Source: Med Decis Making. 2015 Nov;35(8):1023–36. doi: 10.1177/0272989X15585131 (PMC4592957; doi:10.1177/0272989X15585131)
Supplement: Supplementary material [file DS_10.11770272989X15585131_TableB1.pdf]

DOI: 10.1177/0272989X15585131

Table B1: Balance, before and after matching, when applying placebo tests to the RCT control group (practice-level variables)

	Non-participants (n=88,830)	Trial controls (n=1,293)	Matched non-participants (n=1,293)	Standardised difference (variance ratio)	
				Before matching	After matching
Mean practice list size (number of patients per practice ((SD))	9,088 (4,814)	10,041 (5,944)	10,071 (5,758)	17.6 (1.52)	-0.5 (1.07)
Diabetes prevalence	5.7	5.6	5.7	-2.8	-5.5
COPD prevalence	1.6	1.6	1.6	-1.6	-0.5
Heart failure prevalence	0.8	0.8	0.8	12.7	-3.0
Mean socioeconomic deprivation score (SD)	23.8 (9.9)	26.9 (9.7)	26.5 (10.4)	32.0 (0.96)	3.9 (0.87)

Note: Weighted by the sample size for each practice.
