# Supplementary material for: An Approach to Assess Generalizability in Comparative Effectiveness Research: A Case Study of the Whole Systems Demonstrator Cluster Randomized Trial Comparing Telehealth with Usual Care for Patients with Chronic Health Conditions
Source: Med Decis Making. 2015 Nov;35(8):1023–36. doi: 10.1177/0272989X15585131 (PMC4592957; doi:10.1177/0272989X15585131)
Supplement: Supplementary material [file DS_10.11770272989X15585131_TableB2.pdf]

DOI: 10.1177/0272989X15585131

Table B2: Balance, before and after matching, when applying placebo tests to the RCT control group (person-level variables)

	Non-participants (n=88,830)	Trial controls (n=1,293)	Matched non-participants (n=1,293)	Standardised difference (variance ratio)	
				Before matching	After matching
Mean age in years (SD)	66.4 (14.3)	70.8 (11.3)	70.8 (11.1)	34.0 (0.63)	-0.5 (1.04)
Female	46.2	40.3	41.1	-11.9	-1.6
COPD	24.7	60.0	60.2	76.4	-0.3
Diabetes	70.7	34.7	35.5	-77.4	-1.6
Heart failure	12.9	35.2	36.7	54.1	-3.1
Mean CM score (SD)	0.16 (0.15)	0.26 (0.20)	0.26 (0.20)	57.2 (1.78)	0.3 (1.00)
Number of distinct medicines					
1 to 4	41.8	28.0	27.9	-29.3	0.2
5 to 9	42.7	45.2	46.2	5.0	-2.2
10 +	7.3	15.9	16.0	27.2	-0.2
Haemoglobin A1c (mean (SD))*	7.37 (1.63)	8.38 (1.74)	8.23 (1.64)	60.0 (1.13)	8.6 (1.13)
Systolic blood pressure†	133.67 16.46)	132.38 (17.57)	133.30 (17.70)	-7.6 (1.14)	-5.2 (0.99)
Diastolic blood pressure‡	75.94 (9.67)	75.14 (10.16)	75.01 (10.62)	-8.1 (1.10)	1.2 (0.92)
Body mass index §	29.66 (6.27)	28.14 (6.08)	28.69 (6.02)	-24.6 (0.94)	-9.1 (1.02)
Smoking status					
Current smoker	17.6	20.0	19.1	6.0	2.1
Ex-smoker	37.9	53.8	49.4	32.3	8.7
Never smoked	44.5	26.2	31.4	-38.9	-11.4
**Prior numbers of health care contacts per head over various periods of time before index date**					
1-360 days					
Emergency admissions	0.21 (0.67)	0.47 (1.07)	0.45 (0.94)	29.0 (2.60)	2.1 (1.30)
Elective admissions	0.29 (0.99)	0.41 (1.03)	0.38 (0.94)	11.2 (1.07)	3.3 (1.19)
Emergency room visits	0.29 (0.88)	0.48 (1.12)	0.43 (0.94)	19.0 (1.63)	5.1 (1.43)
Outpatient attendances	2.01 (4.01)	3.80 (5.44)	3.53 (4.91)	37.4 (1.84)	5.2 (1.22)
Primary care contacts	11.92 (12.06)	14.55 (12.13)	13.85 (11.00)	21.8 (1.01)	6.1 (1.21)
361-720 days					
Emergency admissions	0.18 (0.62)	0.53 (1.09)	0.50 (1.02)	38.9 (3.06)	2.6 (1.13)
Elective admissions	0.27 (0.96)	0.48 (1.26)	0.43 (1.03)	18.8 (1.74)	4.5 (1.48)
Emergency room visits	0.22 (0.78)	0.42 (0.96)	0.38 (0.97)	22.2 (1.52)	3.5 (0.98)
Outpatient attendances	1.71 (3.61)	3.21 (4.70)	3.00 (4.14)	35.7 (1.69)	4.6 (1.29)
Primary care contacts	11.00 (11.39)	13.35 (11.19)	12.76 (10.48)	20.9 (0.96)	5.5 (1.14)

Notes: Data show percentages unless otherwise specified. CM = Combined Model.

* For the diabetes trial subset only (n=272 intervention patients and 272 matched controls).

DOI: 10.1177/0272989X15585131

† n=86,519, 1,263, and 1,254, for the three groups, respectively
‡ n=86,512, 1,263, and 1,254, for the three groups, respectively
§ n=65,552, 1,007, and 882, for the three groups, respectively.
