# Supplementary material for: An Approach to Assess Generalizability in Comparative Effectiveness Research: A Case Study of the Whole Systems Demonstrator Cluster Randomized Trial Comparing Telehealth with Usual Care for Patients with Chronic Health Conditions
Source: Med Decis Making. 2015 Nov;35(8):1023–36. doi: 10.1177/0272989X15585131 (PMC4592957; doi:10.1177/0272989X15585131)
Supplement: Supplementary material [file DS_10.11770272989X15585131_TableB4.pdf]

DOI: 10.1177/0272989X15585131

Table B4: Sensitivity of incidence rate ratio for emergency hospital admissions to alternative model specifications (on matched data set)

Model	Number of adjustment variables, excluding group	Incidence rate ratio (95% confidence interval)
Full model	51	1.22 (1.05 to 1.43)
Parsimonious model	25	1.24 (1.06 to 1.44)
Including interaction terms with age	32	1.22 (1.05 to 1.43)
With site and interaction terms with site	41	1.25 (1.07 to 1.45)
With interaction terms with condition	53	1.27 (1.09 to 1.47)
Predictive risk score	1	1.31 (1.11 to 1.54)
