# Supplementary material for: An Approach to Assess Generalizability in Comparative Effectiveness Research: A Case Study of the Whole Systems Demonstrator Cluster Randomized Trial Comparing Telehealth with Usual Care for Patients with Chronic Health Conditions
Source: Med Decis Making. 2015 Nov;35(8):1023–36. doi: 10.1177/0272989X15585131 (PMC4592957; doi:10.1177/0272989X15585131)
Supplement: Supplementary material [file DS_10.11770272989X15585131_TableB5.pdf]

DOI: 10.1177/0272989X15585131

Table B5: Results of the placebo test (figures are for the incidence rate ratio for the trial controls *vs.* non-participants and 95% confidence intervals, except for mortality, where figures are for the odds ratio)

	Generalized linear modeling	Time series
Emergency admissions per head	1.22 (1.05, 1.43)	1.31 (1.11, 1.54)
Elective admissions per head	0.99 (0.83, 1.18)	0.97 (0.79, 1.19)
Outpatient attendances per head	1.03 (0.94, 1.13)	1.02 (0.93,1.12)
Emergency room visits per head	1.23 (1.07, 1.43)	1.18 (1.00 1.41)
Primary care contacts per head	0.92 (0.87, 0.97)	0.92 (0.87, 0.98)
Mortality	2.17 (1.16, 4.08)	n/a

Note: Time series models were not defined for mortality, as no deaths occurred before the date of enrolment into the trial.
