# Supplementary material for: An Approach to Assess Generalizability in Comparative Effectiveness Research: A Case Study of the Whole Systems Demonstrator Cluster Randomized Trial Comparing Telehealth with Usual Care for Patients with Chronic Health Conditions
Source: Med Decis Making. 2015 Nov;35(8):1023–36. doi: 10.1177/0272989X15585131 (PMC4592957; doi:10.1177/0272989X15585131)
Supplement: Supplementary material [file DS_10.11770272989X15585131_TableB6.pdf]

Table B6: Sensitivity of incidence rate ratio for the number of emergency hospital admissions per head to alternative model specifications for the time series analysis

**Data level**	**Fixed effects**	**Covariance structure**	**Incidence rate ratio (95% confidence interval)**
Quarterly	Quarterly dummies, group membership and interaction term	First order autoregressive	1.31 (1.11 to 1.54)
Quarterly	Quarter expressed as continuous variable, group membership and interaction term	First order autoregressive	1.25 (1.08 to 1.43)
Quarterly	Quarterly dummies, group membership and interaction term	Spatial	1.31 (1.11 to 1.54)
Monthly	Monthly dummies, group membership and interaction term	First order autoregressive	1.25 (1.06 to 1.48)
