# Supplementary material for: An Approach to Assess Generalizability in Comparative Effectiveness Research: A Case Study of the Whole Systems Demonstrator Cluster Randomized Trial Comparing Telehealth with Usual Care for Patients with Chronic Health Conditions
Source: Med Decis Making. 2015 Nov;35(8):1023–36. doi: 10.1177/0272989X15585131 (PMC4592957; doi:10.1177/0272989X15585131)
Supplement: Supplementary material [file DS_10.11770272989X15585131_TableC1.pdf]

DOI: 10.1177/0272989X15585131

Table C1: Balance, before and after matching, in the sensitivity analysis as applied to the RCT intervention group (practice-level variables)

	Non-participants (n=88,830)	Trial intervention patients (n=1,229)	Matched non-participants (n=1,293)	Standardised difference (variance ratio)	
				Before matching	After matching
Mean practice list size (number of patients per practice ((SD))	9,088 (4,814)	9,417 (3,828)	9,265 (3,719)	7.6 (0.63)	4.0 (1.06)
Diabetes prevalence	5.7	5.8	6.0	13.1	-8.9
COPD prevalence	1.6	1.5	1.5	-10.4	-3.2
Heart failure prevalence	0.8	0.8	0.8	21.1	4.8
Mean socioeconomic deprivation score (SD)	23.8 (9.9)	29.2 (11.5)	28.7 (11.2)	50.4 (1.35)	4.5 (1.05)

Note: Weighted by the sample size for each practice.
